# Nitration in cancer signaling

**DOI:** 10.1016/j.redox.2026.104223

**Published:** 2026-05-19

**Authors:** Inge Claassen, Nirbachita Adrita, Kevin B. Chandler, Stephen M. Black, Blaine R. Roberts, Alvaro G. Estevez, Joseph S. Beckman, Maria Clara Franco

**Affiliations:** a Center for Translational Science, Florida International University, Port St. Lucie, Florida, 34987, USA; b Department of Cellular & Molecular Medicine, Herbert Wertheim College of Medicine, Florida International University, Port St. Lucie, Florida, 34987, USA; c Department of Neurology, Emory University School of Medicine, Atlanta, GA, 30322, USA; d Department of Biochemistry, Emory University School of Medicine, Atlanta, GA, 30322, USA; e Department of Biochemistry and Biophysics, Oregon State University, Corvallis, OR, 97331, USA; f Linus Pauling Institute, Oregon State University, Corvallis, OR, 97331, USA

**Keywords:** Cancer, Reactive nitrogen species, Nitric oxide, Peroxynitrite, Cysteine oxidation, Tyrosine nitration, Tumor biology

## Abstract

Oxidative stress arises from an imbalance between the production of reactive oxygen and nitrogen species (ROS and RNS, respectively) and the capacity of cellular antioxidant defenses to neutralize them. In cancer, this imbalance drives pathological remodeling of signaling networks that promote tumor initiation and progression. Among RNS, nitric oxide (·NO) and its highly reactive derivative peroxynitrite (ONOO^−^) are central mediators of redox dysregulation within the tumor microenvironment. These species induce site-specific post-translational modifications (PTMs), most notably protein tyrosine nitration, which can profoundly alter protein structure, function, interaction networks, and turnover, thereby reshaping essential cellular processes. In this review, we examine the molecular mechanisms of oxidative stress with a particular emphasis on nitration-driven protein modifications and their impact on oncogenic signaling. We highlight accumulating evidence that selective nitration of key signaling proteins actively contributes to multiple hallmarks of malignancy. These nitration events promote tumor initiation and growth, aberrant proliferation, migration and metastasis, metabolic reprogramming, angiogenesis, invasion, resistance to apoptosis, and immune evasion through disruption of core signaling pathways, cell-cycle control, and cell-death programs. Collectively, these findings support an emerging paradigm in which nitrated proteins are not merely passive byproducts of oxidative stress but active effectors of tumorigenesis. We discuss the translational implications of this concept, positioning protein tyrosine nitration as a source of mechanistically defined biomarkers and therapeutic vulnerabilities. A deeper understanding of the selectivity, structural consequences, and biological impact of protein tyrosine nitration will be essential for developing innovative precision strategies to modulate redox signaling in cancer and ultimately improve clinical outcomes.

## 1 Introduction

In cancer, chronic inflammation is a well-established driver of tumor initiation and progression and is characterized by sustained production of reactive nitrogen species (RNS) within the tumor microenvironment [[1], [2], [3], [4], [5], [6], [7], [8], [9]]. RNS encompasses nitric oxide (·NO) and a range of reactive species generated downstream of ·NO production, including the highly reactive oxidant peroxynitrite [10,11].

Nitric oxide exerts a wide range of physiological functions, including the regulation of vasodilation and local blood flow, maintenance of endothelial barrier integrity, and modulation of synaptic signaling and plasticity [[12], [13], [14], [15]]. Under pathological conditions, however, increased or dysregulated ·NO production promotes the formation of other RNS, including peroxynitrite, which mediate oxidative, nitrative, and nitrosative reactions targeting proteins, lipids, and nucleic acids, thereby altering macromolecular structure and function [3,7,8,[16], [17], [18], [19], [20], [21], [22], [23], [24], [25], [26]]. S-nitrosation refers to the chemical modification of thiol groups driven by nitric oxide to form S-nitrosothiols (S–NO) and is commonly termed S-nitrosylation when occurring on protein cysteine residues. Importantly, these redox-dependent modifications are not merely cytotoxic byproducts but function as regulatory mechanisms that modulate intracellular signaling pathways, transcriptional programs, and cell fate decisions, ultimately influencing gene expression, cell growth and differentiation, as well as apoptosis and necrosis [16,21,26,27].

Accumulating evidence demonstrates that RNS are key mediators linking inflammation to oncogenesis by promoting oxidation, nitration, and nitrosation of signaling proteins, transcription factors, and enzymes involved in cell survival, proliferation, and genomic stability [16,[28], [29], [30], [31], [32], [33], [34], [35]]. Here, we examine redox-driven modifications of critical proteins that rewire cellular signaling from homeostatic control toward pro-tumorigenic states. We focus on proteins that are selectively targeted by tyrosine nitration and cysteine oxidation with defined functional consequences, thereby providing a biochemical framework linking RNS signaling to cancer cell migration, invasion, progression, and angiogenic remodeling.

Although RNS lead to a range of oxidative modifications in biomolecules, including lipids and proteins, this review focuses on protein tyrosine nitration as a selective and chemically stable post-translational modification capable of encoding sustained functional changes in cancer signaling.

## 2 The role of nitric oxide and peroxynitrite in tumor biology

In tumors, nitric oxide exhibits a concentration-dependent duality in its biological effects. At relatively low concentrations, ·NO promotes tumor growth, proliferation, angiogenesis, and metastasis, whereas higher concentrations have been reported to induce apoptosis and suppress tumor progression [36]. However, in many tumor contexts, ·NO can also inhibit apoptosis by blocking the activation of caspases, including caspase 9 downstream of cytochrome *c* release from mitochondria, thereby promoting cancer cell survival and proliferation [37,38]. Beyond its direct effects on tumor cells, ·NO plays a fundamental role in endothelial cell biology, regulating migration, proliferation, differentiation, and angiogenesis [[39], [40], [41], [42], [43], [44], [45]]. In the tumor microenvironment, ·NO contributes to neovascularization by inducing angiogenic and lymphangiogenic factor expression, upregulating vascular endothelial growth factor (VEGF), and enhancing endothelial responsiveness to VEGF signaling. In parallel, ·NO modulates matrix metalloproteinase (MMP) expression to facilitate extracellular matrix remodeling, promotes epithelial-to-mesenchymal transition (EMT), and supports invasive and metastatic behavior [[46], [47], [48], [49], [50], [51], [52]].

**Fig. 1:**
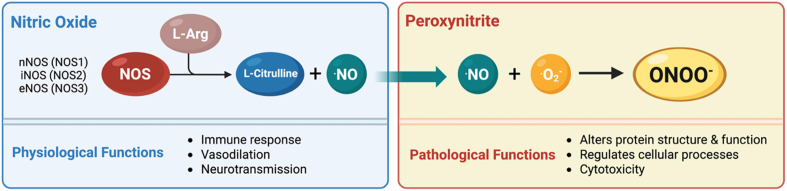
**Production of nitric oxide and peroxynitrite**. Nitric oxide (·NO) is generated from l-arginine (L-Arg) by three nitric oxide synthase (NOS) isoforms, neuronal (nNOS/NOS1), inducible (iNOS/NOS2), and endothelial (eNOS/NOS3). In pathological conditions, ·NO reacts with superoxide (O_2_^.-^) at a diffusion-limited rate, yielding the reactive species peroxynitrite (ONOO^−^).

Many pathogenic outcomes attributed to high ⋅NO levels are, in fact, mediated by peroxynitrite formation. In pathological conditions, ⋅NO reacts with superoxide (O_2_^.-^) at a diffusion-limited rate (5 - 10 × 10^9^ M^−1^s^−1^) to produce secondary species, including peroxynitrite [[53], [54], [55]] (Fig. 1).

Although superoxide dismutases (SODs) efficiently scavenge O_2_^.-^ with an almost diffusion-limited rate of ∼2 × 10^9^ M^−1^s^−1^ [56], peroxynitrite formation remains kinetically favored, as the reaction between ⋅NO and O_2_^.-^ occurs one order of magnitude faster than SOD-catalyzed superoxide dismutation [55]. Once formed, peroxynitrite can modify a variety of amino acids on proteins, including oxidation of sulfur-containing amino acids, cysteine and methionine, and nitration of aromatic amino acids, tryptophan and tyrosine, which, depending on the location of the residue in the protein, can alter protein function. The chemical reactivity of peroxynitrite has been comprehensively described in several excellent review articles [7,16,57,58].

The biological actions of peroxynitrite are likewise concentration and context-dependent: at moderate levels, peroxynitrite can promote cellular processes through selective signaling mechanisms, whereas at high levels it exerts cytotoxic effects, a property exploited by the innate immune system during host defense [59]. Importantly, this duality reflects fundamental differences in peroxynitrite production kinetics and cellular buffering capacity.

Because peroxynitrite is formed by the diffusion-controlled reaction between nitric oxide and superoxide, its biological effects are determined primarily by localized formation rates and spatial confinement rather than absolute steady-state concentrations [32,60]. Consistent with its short half-life and rapid reactions with carbon dioxide, thiols, and metalloproteins, peroxynitrite does not accumulate at high concentrations in biological systems. Its actions are therefore best understood in terms of localized exposure and context-dependent downstream biological outcomes [32].

As mentioned, under pathological conditions, depending on disease context and antioxidant capacity, peroxynitrite production and the resulting oxidative post-translational modifications can selectively regulate signaling pathways without inducing overt cytotoxicity [7,8,61].

## 3 Translating redox chemistry by peroxynitrite into protein function and signaling

The functional consequences of peroxynitrite-mediated modifications must be interpreted within the broader context of competing and supportive post-translational modifications. Peroxynitrite exists in equilibrium between its anionic form (ONOO^−^) and its conjugate acid, peroxynitrous acid (ONOOH), which coexist under physiological conditions and are in rapid acid-base equilibrium with a pKa of approximately 6.8 [20]. At physiological pH values, ONOO^−^ predominates; however, ONOOH is the species responsible for many of the direct two-electron oxidation reactions targeting sulfur-containing amino acids, including cysteine and methionine, as well as tryptophan, with cysteine reacting the fastest [62]. Peroxynitrous acid can also undergo homolytic cleavage to generate hydroxyl radical (⋅OH) and nitrogen dioxide (⋅NO_2_). This reaction occurs with a relatively slow first-order rate constant (∼1.13 M^−1^s^−1^ at pH 7.4 and 37 °C) and therefore competes poorly with faster bimolecular reactions with cellular targets [20]. Instead, under biological conditions, the predominant fate of ONOO^−^ is its rapid reaction with readily available carbon dioxide (CO_2_), forming the transient intermediate nitrosoperoxycarbonate (ONOOCO_2_^−^), which decomposes into carbonate radical (CO_3_^.-^) and nitrogen dioxide (·NO_2_) [[63], [64], [65]]. These secondary radicals are key mediators of ONOO^−^ reactivity in cells and preferentially target amino acids with thiol or aromatic side chains, including cysteine, methionine, tyrosine, and tryptophan.

Biological thiols (RSH/RS^−^) are primarily derived from cysteine amino acids in proteins, and their reactivity is determined by the acid-base equilibrium between the protonated thiol (RSH) and its conjugate base, the thiolate anion (RS^−^) [66]. While protonated thiols are relatively unreactive, the thiolate species can act as strong nucleophiles [66]. Oxidation of the redox-sensitive thiol side chain of cysteine enables it to function as a reversible oxidative switch. Oxidized cysteines can form sulfenic acids (RSOH), which may be further stabilized through disulfide bonds, and act as a functional on-or-off switch. This oxidation state regulates protein activity and downstream signaling, and is at the center of the redox cycling of the thioredoxin- and glutathione-dependent antioxidant systems [67].

**Fig. 2:**
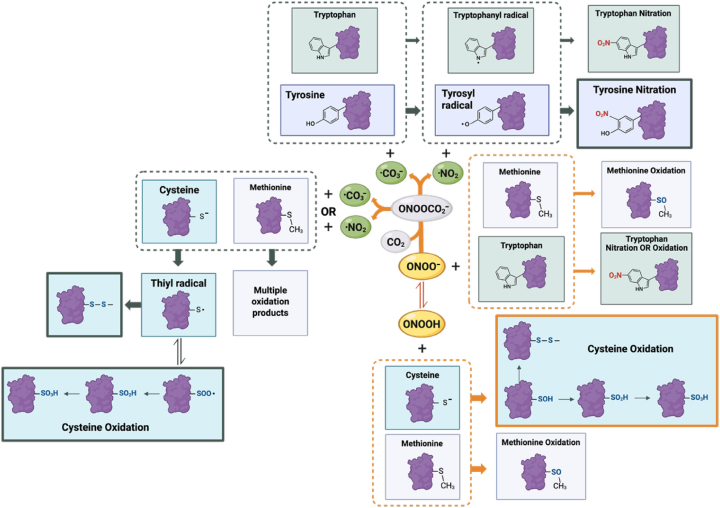
**Peroxynitrite-driven post-translational modifications.** Peroxynitrite (ONOO^−^) and peroxynitrous acid (ONOOH) introduce selective post-translational modifications on multiple amino acid residues. Tyrosine residues are modified by nitration (-NO_2_) to form 3-nitrotyrosine, a stable and largely irreversible modification that can alter protein structure, activity, and protein-protein interactions. Cysteine residues undergo oxidation to sulfenic (-SOH), sulfinic (-SO_2_H), or sulfonic acids (-SO_3_H), as well as disulfide formation (-S-S-) under specific redox conditions. Methionine residues are oxidized to methionine sulfoxide (–SO–CH3), a modification that is reversible through methionine sulfoxide reductase systems. Tryptophan residues undergo oxidative and nitrative modifications, including hydroxylation (oxidation, –OH) and nitrotryptophan formation (-NO_2_), which can impact protein folding and function. Collectively, these peroxynitrite-driven modifications reprogram protein signaling networks and contribute to redox-dependent regulation in disease conditions.

As shown in Fig. 2, cysteine oxidation by ONOOH and ONOO^−^ derivatives can result in both reversible and irreversible changes [68]. The reaction of ONOOH with thiols was the first example of a second-order reaction between peroxynitrite and a biomolecule. The reaction proceeds through a two-electron oxidation mechanism in which ONOOH reacts with thiolate (RS^−^) to yield nitrite (NO_2_^−^) and sulfenic acid [69]. In the absence of stabilizing partners or conditions of excess oxidants, sulfenic acid can be irreversibly oxidized to sulfinic (RSO_2_H) and sulfonic acid (RSO_3_H). Sulfinic acid can be enzymatically reduced by sulfiredoxin; nevertheless, these higher oxidation states often signify oxidative damage and protein dysfunction [70,71].

In addition, as discussed above, thiol oxidation by peroxynitrite can also occur through a competing pathway, particularly relevant in the cellular context, involving CO_3_^.-^ and ⋅NO_2_ derived from ONOO^−^. These radicals oxidize thiols through one-electron reactions, generating thiyl radicals (^⋅^RS) that can react with oxygen to form a thioperoxyl radical (RSOO^⋅^), which can be reduced. The thioperoxyl radical can undergo reduction to sulfinic acid and ultimately result in oxidation to sulfonic acid [66].

Peroxynitrite also oxidizes methionine to methionine sulfoxide (MetO) with a second-order rate constant of approximately 364 M^−1^s^−1^ at pH 7.4 and 37 °C [20,72]. This is an additional marker of oxidative stress [73]. Kinetic analyses indicate that methionine oxidation occurs much faster through ONOOH than through ONOO^−^, with rate constants of approximately 1700 M^−1^s^−1^ for ONOOH compared with only 8.6 M^−1^s^−1^ for ONOO^−^, while the reactions with CO_3_^.-^ and ⋅NO_2_ result in a complex mixture of products [20,74].

In cells, peroxynitrite also reacts with aromatic amino acids tryptophan and tyrosine. For example, in the case of tryptophan residues, ONOOH and ONOO^−^ lead to nitration and oxidation events [75,76]. Of particular relevance to cellular signaling is the nitration of tyrosine residues.

## 4 Mechanistic pathways of protein tyrosine nitration in biological systems

Protein tyrosine nitration arises through enzymatic and non-enzymatic pathways that generate RNS capable of selectively modifying certain tyrosine residues in proteins under distinct biochemical and cellular contexts. The efficiency and selectivity of nitration are strongly influenced by local biochemical conditions, including compartmentalization and redox environment. Tyrosine nitration involves the addition of a nitro group (-NO_2_) to the ortho position adjacent to the hydroxyl group on the aromatic ring of tyrosine [54,77,78], resulting in the formation of 3-nitrotyrosine [54]. The modification can be a result of enzymatic as well as nonenzymatic pathways [3,54,[79], [80], [81], [82], [83], [84], [85], [86], [87], [88], [89], [90], [91]]; however, in biological systems, the predominant relevant chemistry is driven by CO_3_^.-^ and ⋅NO_2_ derived from ONOO^−^. The carbonate radical oxidizes tyrosine to a tyrosyl radical, which subsequently reacts with ⋅NO_2_ to produce nitrotyrosine (Fig. 2). This mechanism predominates in cellular environments where ·NO and O_2_^.-^ are co-produced, such as inflammation and the tumor microenvironment [7,8].

Peroxynitrite reactivity is further modulated by interactions with transition metal centers in metalloproteins and low-molecular-weight complexes. These reactions can proceed through one- or two-electron pathways, generating ·NO_2_ or promoting isomerization of peroxynitrite to nitrate, thereby influencing the balance between nitrative signaling and detoxification. In heme-containing systems, peroxynitrite can generate highly oxidizing intermediates, including oxo-ferryl species, which further contribute to oxidative and nitrative modifications in the presence of suitable substrates [16].

In addition to peroxynitrite-dependent chemistry, enzymatic pathways also contribute to protein nitration. Heme peroxidases, including myeloperoxidase (MPO) and eosinophil peroxidase (EPO), catalyze the oxidation of nitrite (NO_2_^−^) in the presence of hydrogen peroxide (H_2_O_2_), generating nitrogen dioxide and related nitrating species. These reactions are particularly relevant in inflammatory environments enriched in activated neutrophils and eosinophils [92].

Collectively, these pathways demonstrate that protein tyrosine nitration arises from a network of spatially and chemically distinct mechanisms. The relative contribution of each pathway is determined by the local redox environment, availability of reactive intermediates, and cellular context, highlighting the complexity of nitrative signaling in biological systems.

Tyrosine nitration occurs within a complex redox environment where other amino acid residues, specifically methionine and cysteine, can act as alternative targets for oxidation and radical-mediated reactions [93]. Experimental and computational studies in model systems have demonstrated that intramolecular electron transfer between tyrosyl radicals and nearby cysteine residues can influence the outcome of nitrative chemistry, in some cases suppressing tyrosine nitration while promoting the formation of thiyl radicals [94,95]. These findings highlight that the selectivity of protein nitration is not solely dictated by the presence of RNS, but also by local protein structure, residue proximity, and competing redox reactions. These mechanisms have largely been characterized *in vitro*, and their contribution to protein modification in cellular systems remains an area of active investigation. In addition, tyrosine nitration may directly compete with phosphorylation at regulatory residues, further modulating signaling outcomes. Together, these observations support the notion that nitration operates as part of an integrated post-translational modification network.

Nitrotyrosine is a marker of nitric oxide-mediated oxidative inflammatory reactions and is considered a central aspect of peroxynitrite-mediated cytotoxicity [78]. Even moderate changes in peroxynitrite levels over long periods can result in significant oxidation [7,96]. Depending on the location of the tyrosine residue in a protein, nitration can modify protein structure and function, which in turn leads to alterations to the cytoskeleton, production of antigenic epitopes, modifications in enzyme catalytic activities, disruption of cell signaling transduction pathways, dysfunction of critical processes in the cell, and may also induce cell death through apoptosis and/or necrosis [7,78,96]. Although historically associated with cytotoxic injury, peroxynitrite formation and tyrosine nitration are now recognized as mechanisms that generate functionally active nitrated proteins capable of reprogramming critical cellular pathways at the core of disease pathogenesis.

Proteomic analyses have revealed that protein tyrosine nitration is highly selective, targeting specific tyrosine residues on a discrete number of proteins that normally participate in critical cellular processes [77,[97], [98], [99]]. Protein nitration can inhibit or activate protein function, promote degradation, or stabilize altered conformations that drive dysregulation of cellular pathways. For example, nitration of tyrosine 34 in human manganese-superoxide dismutase (MnSOD) leads to its inactivation [100], while the nitration of the small GTPase RhoA activates the protein and stimulates cellular glycolysis [101].

Thus, nitrated proteins are increasingly recognized as a broadly relevant regulatory axis across diverse cancer states, reflecting the pervasive redox imbalance that characterizes the tumor microenvironment. Chronic inflammation, oncogene-driven metabolic stress, hypoxia, and immune-cell infiltration converge to sustain elevated nitric oxide and superoxide production, creating conditions that favor peroxynitrite formation. This localized nitrative stress drives the selective nitration of tyrosine residues on a restricted set of proteins positioned at critical signaling nodes.

Rather than occurring randomly, tyrosine nitration generates functionally distinct protein states that reprogram oncogenic signaling, metabolism, cell fate decisions, and immune interactions across cancer types. In a recent comprehensive review, Griswold-Prenner et al. compiled an extensive database of nearly 1,000 proteins with experimentally confirmed nitration sites, integrating findings accumulated over the past three decades [94]. Many of these proteins that are targets for nitration participate in critical cellular processes dysregulated in cancer, implying that their nitration could have profound implications for oncogenic signaling [20,102,103].

Importantly, although many proteins may become nitrated in pathological conditions, the presence of tyrosine nitration does not inherently imply functional modulation. Defining those proteins with pathological activity within the nitroproteome could open the door to novel mechanistic and therapeutic paradigms.

The following sections examine how peroxynitrite-driven oxidation of critical proteins translates the chemical reactivity of RNS into long-term alterations in cell behavior, with an emphasis on proteins that undergo tyrosine nitration and may also be susceptible to cysteine oxidation by peroxynitrite and nitric oxide. In cancer, this may lead to immune evasion, resistance to apoptosis, increased metastatic potential, and therapeutic resistance. Understanding these downstream modifications bridges the mechanistic gap between oxidative stress and functional dysregulation in tumorigenesis.

## 5 Dysregulation of p53 tumor suppressor function by oxidation

**Fig. 3:**
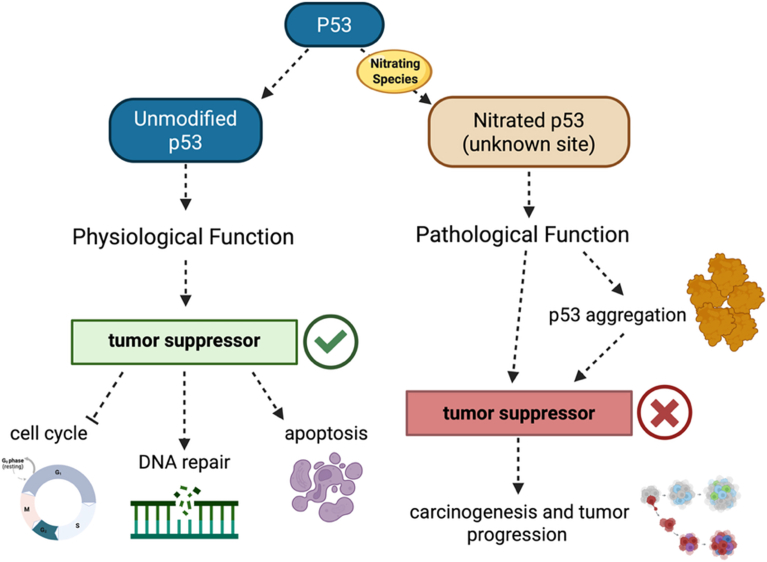
**Tyrosine nitration-mediated inhibition of p53 tumor suppressor activity.** Tyrosine nitration of p53 disrupts p53 DNA-binding capacity, alters protein conformation, inducing aggregation, and interferes with transcriptional activation of p53 target genes involved in cell cycle arrest, DNA repair, and apoptosis. Collectively, this modification has the potential to attenuate p53 tumor suppressor function, facilitating malignant progression under conditions of chronic nitrative stress.

The involvement of p53 in cancer development has been studied for more than 40 years [[104], [105], [106], [107]]. Some studies have shown a correlation between increased ⋅NO production and the selection of mutant p53 cells, which contribute to human carcinogenesis and tumor progression [108]. Camels et al. showed that in human MCF-7 breast cancer cells that express wild-type p53, ⋅NO induces conformational changes in p53, decreasing its binding to DNA and thus impairing the tumor suppressor activity [109]. A subsequent study by the same group confirmed that exposure of p53 to high concentrations of the ^⋅^NO donor S*-*nitrosoglutathione leads to tyrosine nitration and causes aggregation and loss of p53-specific DNA binding, potentially playing a role in carcinogenesis by impairing the functions of this tumor-suppressor protein [109,110] (Fig. 3).

Interestingly, nitrated p53 is detected in human glioblastoma, the most aggressive brain tumor, and its effects are similar to those observed following ⋅NO exposure to recombinant protein or cells *in vitro* [111,112]. This posttranslational modification could contribute to the inactivation of p53 tumor suppressor function in many oxidative diseases, including cancer.

In a more recent study, the authors found p53 exclusively nitrated on Y327 following exposure to low ⋅NO concentrations by incubation with DETA NONOate in MCF7 (breast cancer) and Saos-2 (osteosarcoma) cells. This site-specific nitration promotes p53 oligomerization, nuclear accumulation, and, interestingly, increased DNA-binding activity, but the transcriptional response downstream of nitrated p53 significantly differs from that triggered by DNA damage [113].

DNA-binding of p53 is also regulated by oxidation of C275, C277, C141, and C182. Reversible cysteine oxidation, including sulfenylation and disulfide formation, reduces sequence-specific DNA binding and remodels protein-protein interactions [[114], [115], [116], [117]]. Similarly, S-nitrosation on critical cysteine residues, C242 and C277, destabilizes p53 structure and impairs DNA binding, an important mechanism by which nitrosative stress alters p53-mediated transcriptional regulation in melanoma cells, and may represent a mechanism of p53 inactivation in tumors lacking mutations in *TP53*, the gene that codes for p53 [118,119].

p53 activity is regulated by a complex network of PTMs, including phosphorylation and acetylation, which play major roles in regulating its stability and transcriptional activity under conditions of stress [[120], [121], [122], [123]]. In this context, the functional consequences of tyrosine nitration and S-nitrosation must be interpreted within a broader regulatory framework, where different modifications may coexist and interact to regulate p53 activity. Thus, while nitration and nitrosation have been shown to alter p53 conformation and DNA-binding properties under specific conditions, its contribution to p53 regulation *in vivo* likely reflects an integration of redox-dependent and canonical signaling pathways.

## 6 Oxidation-dependent reprogramming of HER signaling

The human epidermal growth factor receptor (HER) family signals through core pathways, including the MAPKs, JAK/STAT, and PI3K pathways, to regulate cell proliferation, differentiation, migration, and apoptosis [124]. These receptors are often overexpressed, amplified, or mutated in cancer [125]. For example, HER2 receptor amplification is frequently observed in ovarian, bladder, breast, and non-small-cell lung carcinoma, as well as several other tumor types [125]. HER2 is an established therapeutic target, particularly in breast and gastric cancers, where HER2 is overexpressed [126]. Neuregulin-1 (NRG1), a trophic factor containing an epidermal growth factor (EGF)-like domain, signals by activating HER2 [127,128]. NRG1-HER2 signaling is involved in many processes of neural development, including proliferation of neuronal progenitors, neuron migration and survival, glial development and myelination, axon guidance, and synapse formation [127].

**Fig. 4:**
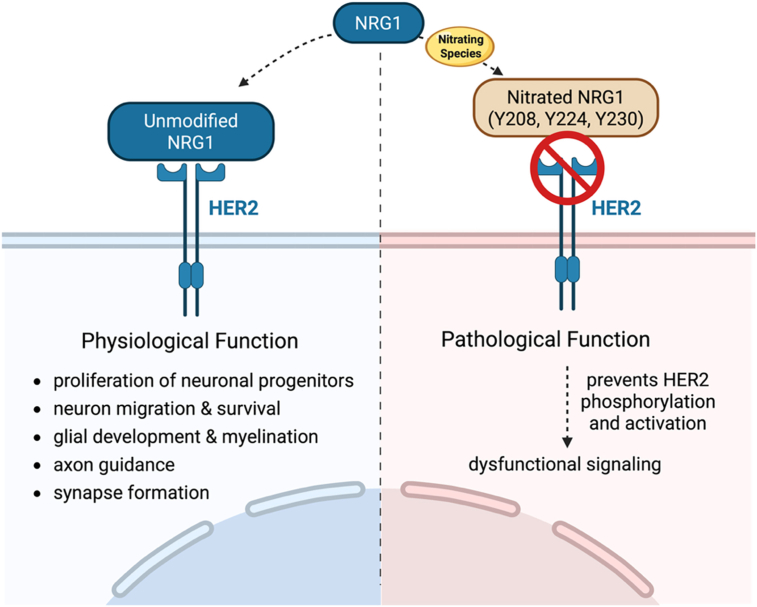
**Nitration of NRG1 disrupts HER2 signaling**. The NRG1/HER2 pathway regulates cell proliferation, differentiation, migration, cell motility, and apoptosis, and is relevant to many cancers. Nitration of NRG1 impairs its binding to HER2, dysregulating downstream signaling, with implications for tumor progression and therapeutic response.

Notably, nitration of NRG1 was shown to decrease binding to HER2. In the lung cancer cell line A549, cytokine stimulation induces iNOS expression, subsequently leading to nitration of NRG1 at Y208, Y224, and Y230. Nitration of NRG1 results in the loss of its ability to phosphorylate, bind, and activate HER2 [128]. Importantly, the redox regulation of HER family signaling is complex and may involve distinct and potentially opposing mechanisms. While nitration of the ligand NRG1 impairs its ability to activate HER2, the receptors themselves, including HER2, EGFR, and Src family kinases, contain redox-sensitive cysteine residues that are critical for their activation. Oxidation or S-nitrosation of these thiol groups has been shown to modulate kinase activity and signaling output [93,129]. In the case of EGFR, cysteine oxidation has been reported to enhance receptor activity [130]. This suggests that under conditions of nitrative or nitrosative stress, differential modification of ligands versus receptors may produce opposing functional outcomes. Thus, redox-dependent modifications may simultaneously reduce ligand-driven signaling while enhancing receptor intrinsic activity, highlighting a context-dependent dichotomy in the regulation of HER family pathways. These findings suggest that nitrative stress can directly reprogram HER2 signaling, although the net outcome likely reflects a balance between inhibitory ligand modifications and activating redox-dependent changes within the receptor itself. (Fig. 4).

The core pathways activated downstream of the HER family can also be regulated by tyrosine nitration and cysteine oxidation, including the mitogen-activated protein kinases (MAPKs) pathway. MAPKs are a family of serine/threonine kinases that are activated by diverse extracellular stimuli and are central regulators of cellular signaling. The MAPK family consists of four major cascades: extracellular signal-regulated kinases 1/2 (ERK1/2), p38 MAPK, c-Jun N-terminal kinase (JNK, also known as stress-activated protein kinase), and ERK5 [131].

Accumulating evidence indicates that tyrosine nitration modulates MAPK signaling in a context-dependent manner, particularly under inflammatory and tumor-associated redox stress. For example, kinases such as ERK1/2, JNK, p38, and ERK5 contain functionally important tyrosine residues within their activation loops, docking interfaces, and regulatory regions [132]. Nitration of these critical tyrosine residues located in close structural proximity to the phosphorylation site could differentially regulate kinase activity and protein stability. An example of this potential regulation is provided by the oxidation of ERK1/2 in stress conditions. Y210 in ERK1 was found nitrated in serum-starved HEK293 cells and after treatment of the recombinant protein with peroxynitrite, which targets the protein for proteasomal degradation, thereby providing a mechanism to downregulate ERK signaling [133]. Similarly, S-nitrosation, potentially at C183, suppresses ERK1/2 phosphorylation and induces apoptosis in MCF-7 cells [134].

Given that ERK signaling is aberrantly activated across numerous cancers, these findings collectively support the concept that redox-dependent modifications, including tyrosine nitration and S-nitrosation, may represent actionable mechanisms to terminate ERK-driven survival and proliferative signaling in tumor cells.

**Fig. 5:**
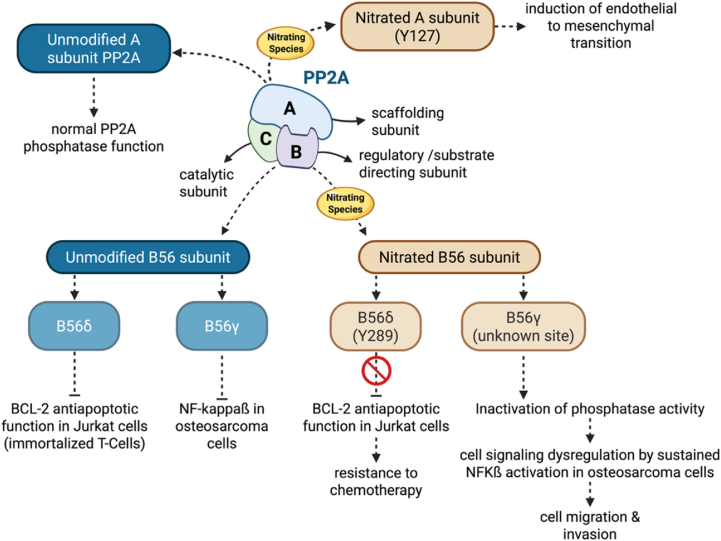
**Nitration of PP2A subunits differentially affects downstream signaling in cancer cells.** Protein phosphatase 2A (PP2A) is a heterotrimeric enzyme composed of a scaffolding A subunit, a regulatory B subunit, and a catalytic C subunit. Both the A and B subunits are targets of tyrosine nitration, leading to dysregulation of downstream signaling pathways relevant to tumor biology. Nitration of the regulatory B subunits B56δ at Y279 and subunit B56γ at an unknown site disrupts their interaction with client proteins. Specifically, nitration of B56δ results in loss of PP2A-mediated inhibition of the anti-apoptotic protein BCL-2, whereas nitration of B56γ promotes sustained NF-κB activation, driving increased cell migration and invasion. In addition, nitration of the scaffolding A subunit at Y127 has been shown to induce endothelial-to-mesenchymal transition.

In addition to regulating kinase activity, tyrosine nitration can also affect cellular phosphatase activity. Protein phosphatase 2A (PP2A) is an important and ubiquitously expressed phosphatase responsible for most phospho-serine and phospho-threonine dephosphorylation, including dephosphorylation of MAPK family members [135,136]. PP2A is a heterotrimer comprised of a catalytic subunit (PP2AC), a scaffolding A subunit (PP2AA), and one of several different isoforms of the regulatory B subunit [126]. Nitration of both the regulatory and catalytic subunits of PP2A has been observed in diverse cellular contexts, and its functional consequences are summarized in Fig. 5.

B56delta (B56δ) is one of the PP2A regulatory subunits that regulates substrate specificity [137]. B-cell lymphoma 2 (BCL2), an overexpressed protein in hematopoietic malignancies, is one of the PP2A-B56δ substrates. BCL2 antiapoptotic activity is regulated by serine S70 phosphorylation, a site that is normally dephosphorylated by PP2A-B56δ to inhibit BCL2 [137,138]. B56δ becomes nitrated on tyrosine Y289 in conditions of increased oxidative stress in Jurkat cells, an immortalized line of human T lymphocytes. B56δ nitrated on Y289 still binds efficiently to Ser70-phosphorylated BCL2; however, the modification inhibits the recruitment of the PP2A catalytic core (A and C subunits), preventing BCL2 dephosphorylation. Hyperphosphorylated BCL2 exerts an anti-apoptotic activity, leading to resistance to chemotherapy-induced apoptosis [139].

In addition, B56gamma (B56γ), a substrate-binding regulatory subunit of PP2A, undergoes nitration in human osteosarcoma U2OS cells, leading to the inactivation of PP2A. PP2A inhibition in these cancer cells triggers sustained NF-kappaβ activation, which enhances cell migration and invasion [140]. The specific sites of B56γ nitration remain to be established.

The catalytic subunit of PP2A is also a target of tyrosine nitration. Nitration of PP2Ac at Y127 has been shown to activate PP2A and induce an endothelial-to-mesenchymal transition in endothelial cells [141], a phenotype with important implications for tumor growth, vascular remodeling, and resistance to cancer therapy [142] (Fig. 5).

In the context of PP2A regulation, the functional consequences of tyrosine nitration may also reflect the interplay between different redox-dependent modifications, rather than a single dominant chemical event.

The downstream pathway involving janus kinase (JAK) and signal transducer and activator of transcription (STAT) can also be dysregulated by tyrosine nitration and cysteine oxidation. The JAK/STAT pathway plays a crucial role in cancer progression by regulating key cellular signaling pathways. In particular, the JNK2/STAT pathway is one of the key signaling cascades that transmit signals from the extracellular environment to the nucleus, downstream of cytokine and growth factors. This pathway is tightly regulated and plays an essential role in processes such as cell proliferation, differentiation, and death [143].

**Fig. 6:**
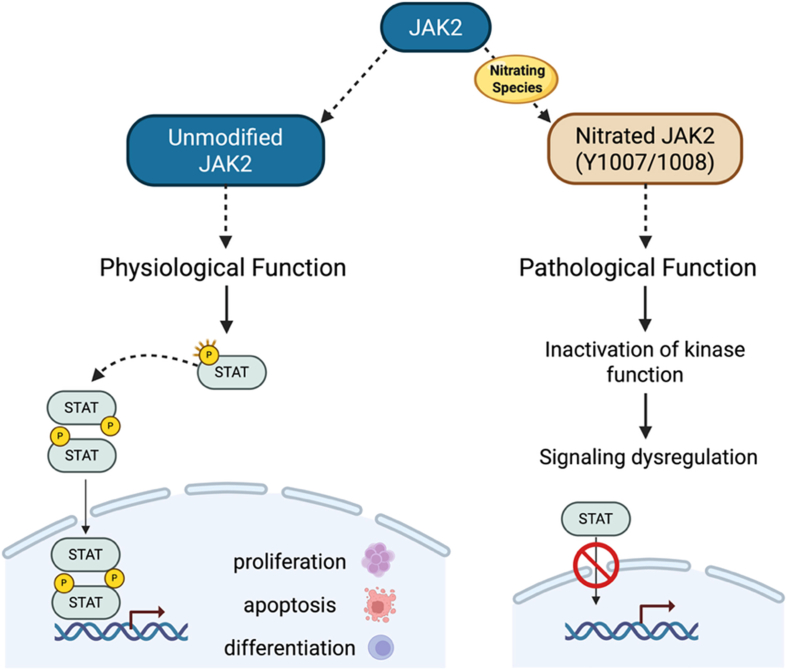
**Nitration of JAK2 affects downstream signaling.** Nitration of JAK2 at Y1007 or Y1008 prevents phosphorylation at Y1007, thereby blocking activation of its kinase activity and dysregulating downstream signaling by the transcription factor STAT.

In certain conditions, JNK2 is a target of nitration, which switches off downstream signaling. For example, treatment with growth hormone induces nitration of JAK2 at Y1007/1008 *in vivo,* assessed in bovine liver, and *in vitro* in cultured porcine hepatocytes. Similarly, a proinflammatory challenge triggered by lipopolysaccharide (LPS) treatment in calves induces nitration of JAK2 at Y1007/1008 in the liver [144,145]. These residues are typically phosphorylated to activate JAK2, and nitration at Y1007 can block further phosphorylation of Y1007 [145], suggesting that inflammatory states regulate nitration and, thus, JAK2 activation, which could be of particular relevance in cancer (Fig. 6).

JAK2 also contains a cysteine-based redox switch at C866 and C917 in which reversible oxidation inactivates the kinase, thereby attenuating cellular responsiveness to JAK-coupled cytokines under conditions of oxidative stress [146]. This redox-sensitive switch predicts that oxidants, including RNS, can impair the response of cells to JAK-coupled cytokines under inflammatory conditions. Consistent with this model, exposure to nitric oxide has been shown to decrease JAK2 signaling, resulting in impaired downstream STAT phosphorylation and reduced transcriptional responses to cytokine stimulation, thereby limiting JAK2-STAT-dependent programs that regulate cell growth, survival, and inflammatory signaling [147].

Extending these observations to downstream effectors, S-nitrosation of STAT3 at C259 in S-nitrosoglutathione (GSNO) stimulates microglia, or in microglia exposed to endogenous ⋅NO via inducible nitric oxide synthase (iNOS), inhibits phosphorylation at Y705, thereby inactivating STAT3 and suppressing downstream proliferative signaling [148]. Together, these findings highlight redox-dependent regulation of the JAK2–STAT axis as a critical mechanism by which RNS reprogram cytokine signaling and suggest that oxidized STAT3 may represent a therapeutically exploitable vulnerability in cancer.

Given that STAT3 activation is tightly regulated by phosphorylation at Y705, the impact of tyrosine nitration at or near regulatory residues is likely to be context-dependent and modulatory rather than a primary driver of activation. Under inflammatory conditions, inflammatory cytokines, like IL-6, act as drivers of iNOS expression, increasing local production of RNS and creating a potential feedback environment in which nitrative modifications may influence STAT3 signaling [[149], [150], [151], [152]]. Tyrosine nitration may interfere with phosphorylation-dependent activation by modifying residues within or proximal to kinase recognition motifs, thereby competing with or sterically hindering phosphorylation events required for STAT3 activation. Nitration may also alter protein structure or interaction with upstream kinases and phosphatases, further fine-tuning pathway activity. However, direct evidence linking site-specific STAT3 nitration to defined functional outcomes remains limited, and the relative contribution of nitration compared to canonical phosphorylation-dependent regulation requires further investigation.

Elucidating how tyrosine nitration and cysteine oxidation alter critical cellular pathways downstream of relevant receptors such as the HER family may therefore provide important insights into how oxidative and nitrative stress reshape cellular signaling in cancer, with potential implications for tumor progression and therapeutic response.

## 7 Regulation of ER proteostasis through tyrosine nitration of PDIA3

Protein disulfide-isomerase a3 (PDIA3), also referred to as Erp57, is a protein disulfide isomerase (PDI) predominantly localized in the endoplasmic reticulum (ER), also found in the nucleus and cell membrane. PDIA3 plays a vital role in ER proteostasis by regulating protein folding of newly synthesized proteins in the ER through disulfide bond formation [[153], [154], [155]]. Notably, PDIA3 was found to be nitrated in muscle from patients with mitochondrial disease, and a prior study showed that exposure of Jurkat cell lysates to peroxynitrite leads to PDIA3 nitration at Y67 and Y100 [156,157]. This oxidative modification could potentially lead to changes in PDIA3 activity and impact the stress response.

**Fig. 7:**
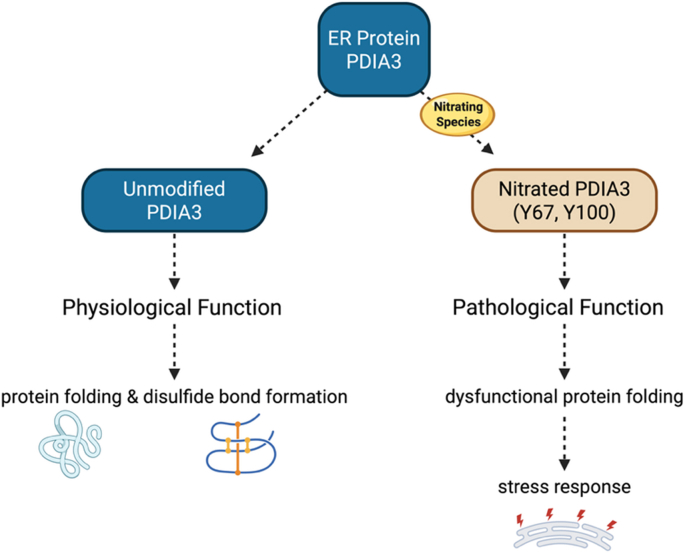
**Nitration of PDIA3 could impact the endoplasmic reticulum (ER) stress response in cancer.** Nitration of the ER molecular chaperone PDIA3 at Y67 and Y100 may alter its binding to clients and chaperone functions, leading to dysfunctional protein folding activity and activation of the endoplasmic reticulum ER stress response.

Chronic ER stress is a defining feature of cancer, conferring adaptive advantages that support tumor survival and malignant progression under unfavorable conditions [154]. PDIA3 is overexpressed across most cancer types and is especially expressed in malignant cells [155]. Elevated PDIA3 has been associated with the extracellular accumulation of proteins that are correlated with aggressive breast cancers [158]. A single-cell sequencing analysis also found that PDIA3 expression is closely associated with cellular processes related to cell communication, metabolism, and epigenetic changes [159]. In diffuse gliomas, increased expression of PDIA3 correlates with poor patient survival and plays a critical role in tumor progression [160]. A separate study by Chiavari et al. reported that overexpression of PDIA3 correlates with a ∼55% reduction in the overall survival of glioma patients [161]. Consistent with these clinical observations, Tu et al. reported that the knockdown of PDIA3 significantly decreases the proliferative and invasive profile of glioma cells [155]. ER stress is a hallmark of cancer [154]; thus, these oxidative modifications could regulate PDIA3 chaperone activity, sustaining the ER stress response. The role of tyrosine nitration in regulating PDIA3 activity and the stress response in cancer remains to be established and warrants further investigation (Fig. 7).

## 8 Metabolic reprogramming driven by tyrosine nitration of the molecular chaperone Hsp90

The 90-kDa heat-shock protein (Hsp90) is a highly conserved and abundant member of the molecular chaperone family in all eukaryotic cells [[162], [163], [164]]. *In vivo*, Hsp90 plays a key role in chaperoning a vast array of client proteins, facilitating folding and stabilization of client proteins, remodeling, and/or maturation [165,166], resulting in conformational changes that can activate or inactivate proteins that are involved in signal transduction pathways and the cell cycle [[162], [163], [164],[167], [168], [169]]. Hsp90 has over 300 known client proteins involved in multiple cellular processes, including kinases and transcription factors [[170], [171], [172]]. Thus, Hsp90 plays a critical role in the regulation of cellular homeostasis, the response to stress [167,169,173,174], as well as the regulation of cell survival and cell death [[170], [171], [172]]. For many clients, Hsp90 chaperone activity is sustained by ATP binding to a pocket in the Hsp90 N-terminal domain, followed by ATP hydrolysis [175]. In mammalian cells, two cytosolic isoforms of Hsp90 are expressed, the inducible Hsp90α and the constitutively expressed Hsp90β [171]. Tyrosine nitration can significantly alter Hsp90 structure and function.

Hsp90β is subject to selective nitration at several tyrosine residues. Hsp90β is nitrated on tyrosine 33 (Y33), 56 (Y56), 276 (Y276), 484 (Y484), and 596 (Y596) [176,177]. Nitration at Y33 and Y56 (Y38 and Y61 in the Hsp90α isoform) induces distinct pathological functions in Hsp90.

Hsp90 is endogenously nitrated in tumors that develop in the genetic disease Neurofibromatosis type 2-related schwannomatosis (NF2-SWN). NF2-SWN is characterized by the development of bilateral vestibular schwannomas and additional nervous system tumors driven by mutations in the *NF2* gene, which encodes the tumor suppressor merlin [177,178]. Loss of merlin function disrupts redox homeostasis by increasing nitric oxide production while decreasing MnSOD-mediated superoxide clearance, resulting in elevated peroxynitrite formation and downstream protein tyrosine nitration [177,178]. Importantly, preventing tyrosine nitration using several complementary approaches markedly reduces NF2-SWN schwannoma cell viability, revealing the existence of one or more nitrated proteins that selectively support tumor cell survival and proliferation [177,178]. In NF2-SWN schwannoma cells, peroxynitrite-driven tyrosine nitration also promotes a metabolic rewiring, characterized by suppressed mitochondrial oxidative phosphorylation and increased glycolysis and glutaminolysis, hallmarks of the Warburg effect [[177], [178], [179], [180]]. This metabolic shift is regulated, at least in part, by site-specific nitrated Hsp90. Nitration of Hsp90 at Y33 suppresses mitochondrial activity, whereas nitration at Y56 enhances glycolytic flux through activation of the P2X7 receptor (P2X7R), collectively supporting tumor cell proliferation [177].

Notably, nitrated Hsp90 gain-of-function triggers different signaling pathways and cellular outcomes depending on the cell type. In highly energetic motor neurons, nitration of Hsp90 at Y33 and Y56 drives mitochondrial dysfunction and apoptotic cell death, thereby contributing to neurodegeneration rather than proliferation [176]. In motor neurons, nitrated Hsp90 activates the P2X7R, which in turn leads to activation of the Fas death receptor. These pathogenic effects have direct implications for disorders such as amyotrophic lateral sclerosis (ALS) and spinal cord injury [176].

Interestingly, in PC12 cells, a neuron-like culture model, when differentiated, Hsp90_NY56_ induces cell death through activation of P2X7R, but the downstream signaling differs from that described in motor neurons, simultaneously activating the p38 and JNK pathways, while inhibiting the PI3K/Akt pathway [181]. On the other hand, Hsp90_NY33_ does not induce cell death but, instead, forms a mitochondrial complex that decreases mitochondrial activity [181,182].

**Fig. 8:**
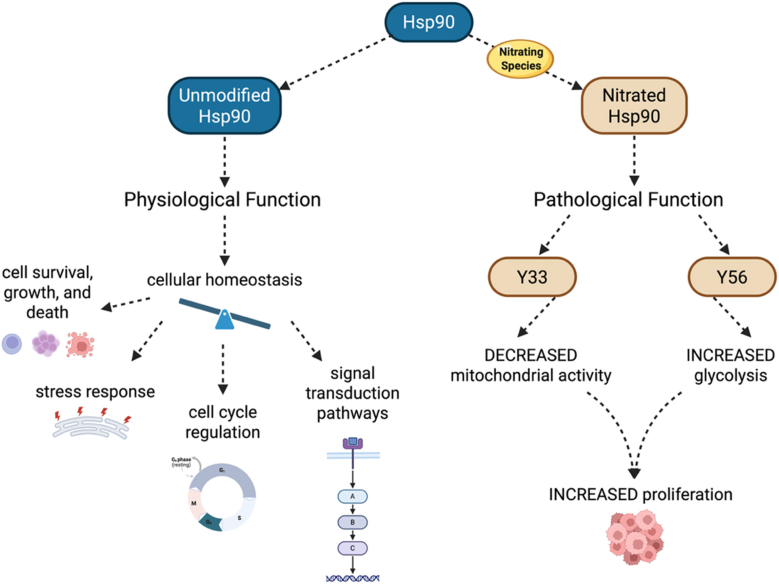
**Tyrosine nitration converts Hsp90 into a metabolic switch in tumor cells.** Hsp90 is a ubiquitous molecular chaperone essential for cellular homeostasis. In tumor cells, tyrosine nitration of Hsp90 at Y33 or Y56 confers distinct metabolic functions. Nitration at Y33 suppresses mitochondrial metabolism, whereas nitration at Y56 promotes glycolytic reprogramming to support tumor cell proliferation.

The emergence of these distinct pathological functions likely reflects nitration-induced, site-specific structural alterations in Hsp90. Indeed, biochemical and structural studies have demonstrated that nitration at Y33 and Y56 produces discrete conformational and functional changes that confer biological activities not observed in its unmodified form [183]. Collectively, these findings support a model in which site-specific nitration remodels Hsp90 structure to generate novel disease-driving functions, potentially by altering its interactome. Elucidating the high-resolution structures of these nitrated Hsp90 variants may therefore uncover new therapeutic strategies to selectively target the nitrated variants with pathological function while preserving normal chaperone function (Fig. 8).

Interestingly, both Y33 and Y56 have been described as targets of phosphorylation. Phosphorylation at Y33 (Y24 in yeast) by Swe1 in the nucleus, prominently during the S phase of the cell cycle in yeasts, and subsequent cytosolic translocation and ubiquitination lead to Hsp90 proteasomal degradation, suggesting that phosphorylation may act as a “switch off” mechanism, at least in yeast [184]. In contrast, tagged recombinant human Hsp90 with nitrotyrosine at position 33 was detected in schwannoma cells 48 h after intracellular delivery, suggesting that Y33 nitration does not trigger Hsp90 degradation [177]. In yeast, expression of Hsp90 in which Y24 was replaced by phosphorylation-resistant phenylalanine made the cells insensitive to G2/M checkpoint arrest and caused premature nuclear division [185], suggesting that there may be a regulatory interplay between nitration and phosphorylation in the regulation of cell cycle progression, cell growth and proliferation.

Tyrosine 56 has also been identified as a phosphorylation site across multiple cancer types in diverse proteomic and phosphoproteomic studies [[186], [187], [188], [189], [190], [191], [192]]. However, the functional significance of this modification in the cellular context has not yet been defined.

In addition to tyrosine nitration, Hsp90 can undergo other oxidative post-translational modifications, including S-nitrosation [193]. S-nitrosation of Hsp90 inhibits its ATPase activity and impairs its regulatory interaction with endothelial nitric oxide synthase (eNOS), establishing a negative feedback loop that limits ⋅NO production [193]. In endothelial cells, S-nitrosation of Hsp90 at C521 serves as a conformational switch that increases binding to the co-chaperone CDC37, a process implicated in the exacerbation of atherosclerosis [194].

CDC37 is a kinase-targeting co-chaperone of the Hsp90 complex that plays a central role in cancer biology by stabilizing a broad spectrum of mutated or overexpressed oncogenic kinases [195], suggesting that S-nitrosation of Hsp90 may be relevant to cancer progression. Whether S-nitrosation and tyrosine nitration of Hsp90 are mechanistically linked or act cooperatively to enhance cancer cell proliferation remains an important unanswered question.

## 9 Nitrative stress promotes tumor immune evasion

The tumor microenvironment consists of cancer-associated fibroblasts, endothelial cells, pericytes, diverse immune cell types, and various additional tissue-resident cell types, blood vessels, and signaling molecules, and plays a crucial role in influencing tumor growth, metastasis potential, and therapeutic response [196,197]. This microenvironment impairs T lymphocyte function, with tumor-infiltrating lymphocytes (TILs) often exhibiting defects in signal transduction and cytotoxic activity [198]. In agreement, there are elevated nitrotyrosine levels present in tumor tissues and TILs, and RNS impair T cell-mediated immunity in the tumor microenvironment [199,200] [201]. In this context, peroxynitrite can be produced by tumor-associated myeloid cells, including myeloid-derived suppressor cells (MDSCs), leading to T cell receptor (TCR) nitration and impaired T cell responses to tumor antigens [200,202,203]. Nagaraj et al. demonstrated that MDSCs induce tyrosine nitration within the TCR-CD8 complex, thereby disrupting the binding of peptide-major histocompatibility complex (pMHC) dimers to CD8^+^ T cells and preventing antigen-specific T-cell responses [200].

In human prostate cancer, Bronte et al. reported that ONOO^−^ is generated through the interplay between the l-arginine metabolizing enzymes arginase (ARG) and NOS and causes TIL unresponsiveness to stimuli, bypassing the TCR [199]. The simultaneous targeting of ARG and NOS reduces protein nitration and restores TIL function, identifying RNS-driven nitrative stress as a central mechanism of tumor-induced immune dysfunction [199]. Using a combination of cell culture assays and *in vivo* assays in three different models, including a colon carcinoma (C26GM), a mouse thymoma engineered to express OVA as a tumor antigen (EG7-OVA), and a prostate cancer spontaneously arising in transgenic adenocarcinoma of the mouse prostate (TRAMP) mouse, Molon et al. further demonstrated that intratumoral RNS production leads to nitration of the CCL2 chemokine, resulting in TIL impairment and the trapping of tumor-specific T cells in the peritumoral stroma surrounding cancer cells [201]. However, the specific mechanism of action and the identity of the nitrated residues have not been established.

**Fig. 9:**
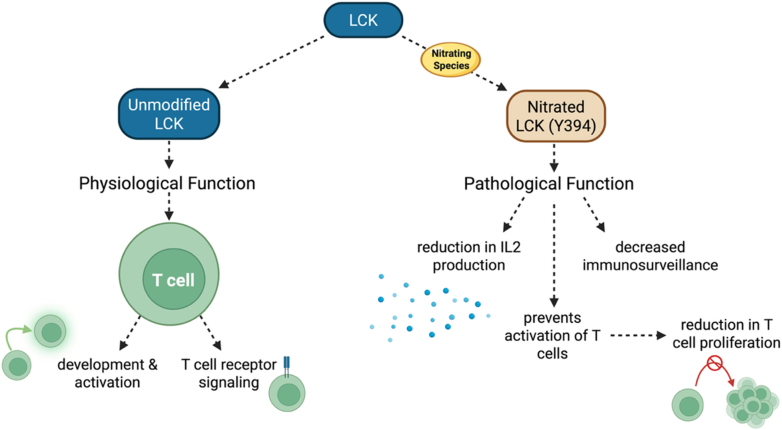
**Nitration of LCK inhibits T cell activation and proliferation.** LCK is fundamental for the development and activation of T cells. LCK is nitrated in tumor-infiltrating lymphocytes, leading to decreased T-cell activation, proliferation, and IL-2 production.

Another clear example of RNS regulation of the immune response in cancer is the nitration of the lymphocyte-specific cytoplasmic protein-tyrosine kinase (LCK). LCK is fundamental for the development and activation of T cells, as well as T cell receptor signaling [204,205]. A chemical derivation method has been developed that can detect nitropeptides from tumor-infiltrating T cells obtained from lung carcinoma and prostate cancer models. Using this method, the authors detected nitration of LCK at tyrosine Y394. Nitration inhibits LCK activity and T cell activation, leading to reduced interleukin 2 production and proliferation of tumor-infiltrating T-lymphocytes [204]. These findings identify RNS-driven tyrosine nitration of LCK as a direct molecular mechanism by which MDSCs suppress T-cell activation to decrease antitumor immune responses (Fig. 9).

Peroxynitrite further contributes to TILs dysfunction by inducing T cell apoptosis through multiple mechanisms, including inhibition of protein tyrosine phosphorylation via tyrosine nitration and modification of mitochondrial channels by nitration of the voltage-dependent anion channel [206] [97].

**Fig. 10:**
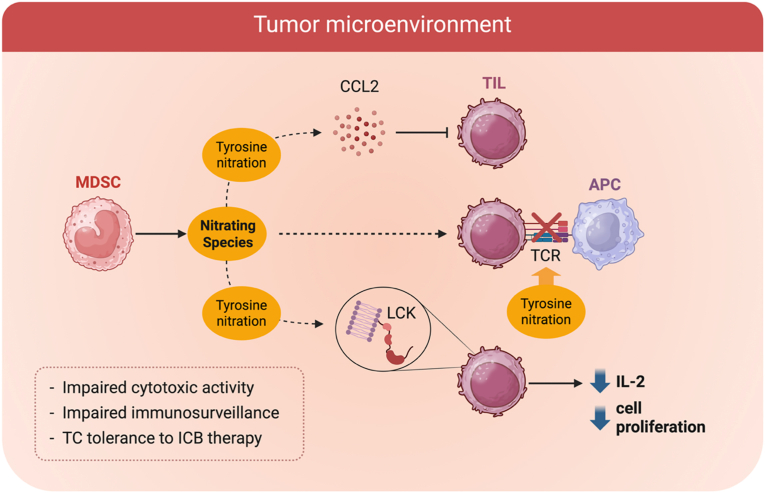
**Regulation of the tumor immune response by peroxynitrite and tyrosine nitration**. In the tumor microenvironment, myeloid-derived suppressor cells (MDSC) produce nitrating species, leading to nitration of key proteins that regulate the activation of tumor-infiltrating lymphocytes (TILs). Nitration of the CD8 T cell receptor (TCR) and the kinase LCK in TILs, and nitration of the CCL2 chemokine result in impaired cytotoxic activity, immunosurveillance, and T cell (TC) tolerance to immune checkpoint blockade (ICB) immunotherapy.

From a therapeutic perspective, activated MDSC can induce T cell tolerance to immune checkpoint blockade (ICB) therapy through the secretion of RNS, resulting in tyrosine nitration of proteins involved in T cell function. Thus, targeting RNS production could be leveraged to regulate immune evasion, as preconditioning tumors with compounds that prevent tyrosine nitration restores CTL function and enhances the antitumor activity, supporting the potential of this approach in cancer immunotherapy [201,204] (Fig. 10).

These findings emphasize how RNS impair T cell function within the tumor microenvironment through targeted nitration of proteins, ultimately promoting immune evasion. Targeting RNS-mediated mechanisms and nitrated proteins may present a promising strategy for restoring antitumor immunity and enhancing immunotherapy efficacy.

Beyond direct impairment of T cell signaling, protein tyrosine nitration may also influence tumor-immune interactions through the generation of immunogenic neoepitopes. The addition of a nitro group to tyrosine residues can alter peptide structure, charge, and antigen processing, potentially giving rise to modified peptides that are recognized as non-self by the immune system. Nitrated peptides have been shown to modulate antigen presentation and T cell recognition in inflammatory and autoimmune diseases, supporting the concept that nitration can reshape the immunopeptidome [[207], [208], [209]]. In line with this, peroxynitrite-driven modifications within the tumor microenvironment have been shown to alter the antigenic landscape, enabling immune evasion and escape from immunotherapy [210]. Depending on the biological context, these modifications may promote immune evasion by impairing antigen recognition or enhance immunogenicity by generating novel antigenic determinants. In this regard, nitration-derived neoepitopes and nitrated protein exposed on the surface of tumor cells may represent an underexplored class of tumor-associated antigens with potential relevance for emerging immunotherapeutic strategies, including antibody-based approaches and chimeric antigen receptor (CAR)-T cell therapy. Thus, protein nitration represents a potential link between redox dysregulation and immune surveillance, with implications for biomarker development and therapeutic targeting.

## 10 Expanding the cancer nitroproteome: new candidates for functional discovery

In addition to proteins whose nitration has been mechanistically linked to cancer pathology, the nitroproteome database assembled by Griswold-Prenner et al. reveals a substantially broader landscape of proteins that undergo tyrosine nitration, many of which remain poorly characterized in the context of tumor biology [103]. From this curated resource, Table 1 highlights a subset of nitrated proteins whose known cellular functions suggest potential relevance to cancer-associated processes, despite the absence of direct functional studies in oncologic settings. Given the pathological gain-of-function described for other nitrated proteins, as well as the roles of these candidates in related disease contexts, systematic investigation of their nitrated states in cancer may uncover previously unrecognized therapeutic vulnerabilities.

**Table 1:** Additional nitrated proteins with potential roles in cancer.

Protein	Nitration Site	Reference	Physiological Role
Superoxide dismutase [Mn], mitochondrial (EC 1.15.1.1)	58, 69, 217	[211]; [100]; [212]; [213]	Mitochondrial antioxidant enzyme detoxifying superoxide. SOD2 converts superoxide to oxygen and hydrogen peroxide, protecting mitochondrial integrity and regulating redox signaling [211].
14-3-3 protein epsilon (14-3-3E)	8, 20, 131, 214	[156]; [214]	14-3-3 protein epsilon is involved in signal transduction, apoptosis, autophagy, cell cycle regulation, cell proliferation, and immunity [215].
14-3-3 protein eta (Protein AS1)	20, 133	[214]	An adaptor protein involved in signaling pathways. 14-3-3 proteins bind phosphoserine/threonine motifs, modulating activity of client proteins. Plays an important role in regulatory processes, including mitogenic signal transduction, apoptosis, and cell cycle regulation [216].
14-3-3 protein gamma (Protein kinase C inhibitor protein 1) (KCIP-1) [Cleaved into: 14-3-3 protein gamma, N-terminally processed]	133	[214]	An adaptor protein involved in signaling pathways. 14-3-3 proteins bind phosphoserine/threonine motifs, modulating activity of client proteins. Plays an important role in regulatory processes, including mitogenic signal transduction, apoptosis, and cell cycle regulation [216].
14-3-3 protein sigma (Epithelial cell marker protein 1) (Stratifin)	19, 130	[214]	An adaptor protein involved in signaling pathways. 14-3-3 proteins bind phosphoserine/threonine motifs, modulating activity of client proteins. Plays an important role in regulatory processes, including mitogenic signal transduction, apoptosis, and cell cycle regulation [216].
14-3-3 protein theta (14-3-3 protein T-cell) (14-3-3 protein tau) (Protein HS1)	19, 48 104, 128	[214]	An adaptor protein involved in signaling pathways. 14-3-3 proteins bind phosphoserine/threonine motifs, modulating activity of client proteins. Plays an important role in regulatory processes, including mitogenic signal transduction, apoptosis, and cell cycle regulation [216].
14-3-3 protein zeta/delta (Protein kinase C inhibitor protein 1) (KCIP-1)	19, 128	[156]; [214]	An adaptor protein involved in signaling pathways. 14-3-3 proteins bind phosphoserine/threonine motifs, modulating activity of client proteins. Plays an important role in regulatory processes, including mitogenic signal transduction, apoptosis, and cell cycle regulation [216].
ATP synthase membrane subunit K, mitochondrial (ATP synthase membrane subunit DAPIT, mitochondrial) (Diabetes-associated protein in insulin-sensitive tissues) (HCV F-transactivated protein 2) (Up-regulated during skeletal muscle growth protein 5)	10, 18, 22	[156]; [214]	Subunit of mitochondrial ATP synthase. Integral part of ATP synthesis from ADP and phosphate, essential for cellular energy [217].
ATP synthase subunit alpha, mitochondrial (ATP synthase F1 subunit alpha)	337, 440	[218]; [219]	Subunit of mitochondrial ATP synthase. Integral part of ATP synthesis from ADP and phosphate, essential for cellular energy [217].
ATP synthase subunit beta, mitochondrial (EC 7.1.2.2) (ATP synthase F1 subunit beta)	196, 230, 247, 269, 292, 331, 361, 395, 418, 499, 508	[220]; [156]; [221]; [217]; [157]; [214]	Subunit of mitochondrial ATP synthase. Integral part of ATP synthesis from ADP and phosphate, essential for cellular energy [217].
ATP synthase subunit d, mitochondrial (ATPase subunit d) (ATP synthase peripheral stalk subunit d)	57, 86, 115	[217]; [222]; [214]	Subunit of mitochondrial ATP synthase. Integral part of ATP synthesis from ADP and phosphate, essential for cellular energy [217].
ATP synthase subunit epsilon, mitochondrial (ATPase subunit epsilon) (ATP synthase F1 subunit epsilon)	12, 15	[214]	Subunit of mitochondrial ATP synthase. Integral part of ATP synthesis from ADP and phosphate, essential for cellular energy [217].
Cofilin-1 (18 kDa phosphoprotein) (p18) (Cofilin, non-muscle isoform)	68, 140	[156]; [223]; [214]	Actin-binding protein important for cytoskeletal dynamics. Severs actin filaments to regulate turnover, motility, and morphology [224,225].
Cytochrome *b*5 type B (Cytochrome *b*5 outer mitochondrial membrane isoform)	142, 143	[214]	Cytochrome *b*5 type B plays an essential role in electron transport and various metabolic processes. It is essential for maintaining cellular homeostasis and influences the activity of several enzymes [226].
Cytochrome *b*-*c*1 complex subunit 1, mitochondrial (Complex III subunit 1) (Core protein I) (Ubiquinol-cytochrome-c reductase complex core protein 1)	186, 219, 257, 420, 448, 450, 459, 468	[157]; [214]	Intrinsic membrane protein that plays a central role in the mitochondrial respiratory chain. It functions as a proton pump catalyzing the oxidation of ubiquinol (ubihydroquinone) and the reduction of cytochrome *c*. Through a Q-cycle mechanism, it couples electron transfer to the translocation of protons across the inner mitochondrial membrane, generating the proton gradient driving ATP synthesis [227].
Cytochrome *b*-*c*1 complex subunit 2, mitochondrial (Complex III subunit 2) (Core protein II) (Ubiquinol-cytochrome-c reductase complex core protein 2)	71	[214]	Intrinsic membrane protein that plays a central role in the mitochondrial respiratory chain. It functions as a proton pump catalyzing the oxidation of ubiquinol (ubihydroquinone) and the reduction of cytochrome *c*. Through a Q-cycle mechanism, it couples electron transfer to the translocation of protons across the inner mitochondrial membrane, generating the proton gradient driving ATP synthesis [227].
Cytochrome *b*-*c*1 complex subunit 7 (Complex III subunit 7) (Complex III subunit VII) (QP-C) (Ubiquinol-cytochrome c reductase complex 14 kDa protein)	39, 84, 90, 94	[214]	Intrinsic membrane protein that plays a central role in the mitochondrial respiratory chain. It functions as a proton pump catalyzing the oxidation of ubiquinol (ubihydroquinone) and the reduction of cytochrome *c*. Through a Q-cycle mechanism, it couples electron transfer to the translocation of protons across the inner mitochondrial membrane, generating the proton gradient driving ATP synthesis [227].
Cytochrome *c*	74, 98	[228]; [229]; [230]	Cytochrome *c* plays a crucial role in the electron transport chain, is an important component in apoptosis, and detoxifies reactive oxygen species [231].
Cytochrome *c* oxidase subunit 4 isoform 1, mitochondrial (Cytochrome *c* oxidase polypeptide IV) (Cytochrome *c* oxidase subunit IV isoform 1) (COX IV-1)	38	[156]	Cytochrome *c* oxidase is the terminal complex of oxidative phosphorylation, which reduces oxygen to water while pumping protons across the mitochondrial membrane. This generates the gradient that powers ATP synthesis [232].
Cytochrome *c* oxidase subunit 5A, mitochondrial (Cytochrome *c* oxidase polypeptide Va)	123	[214]	Cytochrome *c* oxidase is the terminal complex of oxidative phosphorylation, which reduces oxygen to water while pumping protons across the mitochondrial membrane. This generates the gradient that powers ATP synthesis [232].
Cytochrome *c* oxidase subunit NDUFA4 (Complex I-MLRQ) (CI-MLRQ) (NADH-ubiquinone oxidoreductase MLRQ subunit)	62	[156]	Cytochrome *c* oxidase is the terminal complex of oxidative phosphorylation, which reduces oxygen to water while pumping protons across the mitochondrial membrane. This generates the gradient that powers ATP synthesis [232].
Cytochrome *c*1, heme protein, mitochondrial (EC 7.1.1.8) (Complex III subunit 4) (Complex III subunit IV) (Cytochrome *b*-*c*1 complex subunit 4) (Ubiquinol-cytochrome-c reductase complex cytochrome *c*1 subunit) (Cytochrome *c*-1)	132, 174, 179	[233]; [234]	A multi-functional enzyme involved in the electron transfer as part of the mitochondrial electron transport chain (ETC), it is crucial for the production of energy. It is also fundamental in the progression of apoptosis [235].
Elongation factor 1-alpha 1 (EF-1-alpha-1) (Elongation factor Tu) (EF-Tu) (Eukaryotic elongation factor 1 A-1) (eEF1A-1) (Leukocyte receptor cluster member 7)	29, 85, 86, 141, 167, 177, 254	[156]; [214]	eEF1A delivers aminoacyl-tRNAs to the ribosomal A site. It belongs to the GTPase superfamily and functions through GTP binding and hydrolysis [236].
Elongation factor 1-alpha 2 (EF-1-alpha-2) (Eukaryotic elongation factor 1 A-2) (eEF1A-2) (Statin-S1)	85, 86	[214]	eEF1A delivers aminoacyl-tRNAs to the ribosomal A site. It belongs to the GTPase superfamily and functions through GTP binding and hydrolysis [236].
Elongation factor 1-delta (EF-1-delta) (Antigen NY-CO-4)	26, 238	[156]; [214]	EF-1-delta is a part of the EF-1 protein complex that mediates the elongation step of protein synthesis. It delivers aminoacyl-tRNAs to the ribosomal A site and functions through GTP binding and hydrolysis [237,238].
Elongation factor 2 (EF-2)	265, 434, 443, 457, 634, 639, 671	[156]; [214]	EF-2 plays an essential role in the synthesis of proteins where it catalyzes the translocation of the mRNA and two tRNAs after peptidyl transfer on the ribosome [239].
Elongation factor G, mitochondrial (EF-Gmt) (Elongation factor G 1, mitochondrial) (mEF-G 1) (Elongation factor G1) (hEFG1)	235, 735	[214]	Protein synthesis EF-G is an essential protein in elongation and ribosome recycling. EF-G coordinates the movement of deacylated tRNA and dipeptidyl-tRNA during elongation, assisting the release of deacylated tRNA from the ribosomal E site [240].
Elongation factor Tu, mitochondrial (EF-Tu) (P43)	95, 249, 269	[214]	EF-Tu transports aminoacylated-tRNAs to the ribosome [241].
Elongin-B (EloB) (Elongin 18 kDa subunit) (RNA polymerase II transcription factor SIII subunit B) (SIII p18) (Transcription elongation factor B polypeptide 2)	45	[156]	A subunit of the heterodimeric Elongin BC complex which positively regulates RNA polymerase II elongation factor Elongin A [242,243].
Glutathione reductase, mitochondrial (GR) (GRase) (EC 1.8.1.7)	150, 158	[156]; [244]; [245]	Maintains reduced glutathione pool. Catalyzes NADPH-dependent reduction of GSSG to GSH, vital for antioxidant defense and control of ROS [244].
Glutathione S-transferase omega-1 (GSTO-1) (EC 2.5.1.18) (Glutathione S-transferase omega 1-1) (GSTO 1-1) (Glutathione-dependent dehydroascorbate reductase) (EC 1.8.5.1) (Monomethylarsonic acid reductase) (MMA(V) reductase) (EC 1.20.4.2) (S-(Phenacyl)glutathione reductase) (SPG-R)	82, 108	[156]	GSTO enzymes are involved in cellular defense. GSTOs have proven to be involved in the detoxification of exogenous stressors [246].
Glutathione S-transferase P (EC 2.5.1.18) (GST class-pi) (GSTP1-1)	50	[156]	Detoxification enzyme. GSTs play a fundamental role in detoxification by conjugating harmful metabolites, ROS, and lipid peroxidation products, and protecting cells from oxidative and toxic damage [247].
Glyceraldehyde-3-phosphate dehydrogenase (GAPDH) (EC 1.2.1.12) (Peptidyl-cysteine S-nitrosylase GAPDH) (EC 2.6.99.-)	140, 276, 314, 320	[248]; [97]; [249]; [250]; [99]; [214]	Glycolytic enzyme involved in bioenergetic metabolism and production of ATP. GAPDH is also a moonlighting protein and can function as pro-apoptotic or anti-apoptotic [251]. GAPDH is involved in the cell's response to oxidative stress; involved in cell injury, apoptosis and cell death under conditions of stress [252,253].
GTPase HRas (EC 3.6.5.2) (H-Ras-1) (Ha-Ras) (Transforming protein p21) (c-H-ras) (p21ras) [Cleaved into: GTPase HRas, N-terminally processed]	4, 40, 96, 137, 157	[156]; [254]	Small GTPase. HRas regulates the PI3K pathway, controlling cell growth, division, apoptosis, and differentiation [255].
Heat shock 70 kDa protein 1A (Heat shock 70 kDa protein 1) (HSP70-1) (HSP70.1)/Heat shock 70 kDa protein 1B (Heat shock 70 kDa protein 2) (HSP70-2) (HSP70.2)	371, 545	[256]	ATP-dependent molecular chaperone essential for folding and quality control. Hsp70 binds misfolded proteins, preventing denaturation and aggregation, assisting in degradation or folding [257].
Heat shock 70 kDa protein 4 (HSP70RY) (Heat shock 70-related protein APG-2)	336	[156]	ATP-dependent molecular chaperone essential for folding and quality control. Hsp70 binds misfolded proteins, preventing denaturation and aggregation, assisting in degradation or folding [257].
Heat shock cognate 71 kDa protein (EC 3.6.4.10) (Heat shock 70 kDa protein 8) (Lipopolysaccharide-associated protein 1) (LAP-1) (LPS-associated protein 1)	41, 115, 183, 371	[218]; [223]; [156]; [258]; [259]	ATP-dependent molecular chaperone essential for folding and quality control. Hsp70 binds misfolded proteins, preventing denaturation and aggregation, assisting in degradation or folding [257].
Heat shock protein HSP 90-alpha (EC 3.6.4.10) (Heat shock 86 kDa) (HSP 86) (HSP86) (Lipopolysaccharide-associated protein 2) (LAP-2) (LPS-associated protein 2) (Renal carcinoma antigen NY-REN-38)	197, 216, 309, 313, 438, 465, 466, 627	[223]; [156]; [214]	ATP-dependent molecular chaperone that facilitates folding and stabilization, remodeling, and/or maturation [165,166], resulting in conformational changes that activate or inactivate proteins involved in signal transduction pathways and the cell cycle [[162], [163], [164],[167], [168], [169]]. Hsp90 plays an important role in the regulation of cellular homeostasis, the response to stress [167,169,173,174], as well as the regulation of cell survival and cell death [[170], [171], [172]].
NADH dehydrogenase [ubiquinone] 1 alpha subcomplex assembly factor 2 (B17.2-like) (B17.2L) (Mimitin) (Myc-induced mitochondrial protein) (MMTN) (NDUFA12-like protein)	32, 33, 34, 38	[214]	Essential component of complex I of the respiratory chain, which transfers electrons from NADH to ubiquinone which is associated with proton pumping out of the matrix [260].
NADH dehydrogenase [ubiquinone] 1 alpha subcomplex subunit 10, mitochondrial (Complex I-42kD) (CI-42kD) (NADH-ubiquinone oxidoreductase 42 kDa subunit)	38, 143, 339, 343	[214]	Essential component of complex I of the respiratory chain, which transfers electrons from NADH to ubiquinone which is associated with proton pumping out of the matrix [260].
NADH dehydrogenase [ubiquinone] 1 alpha subcomplex subunit 12 (13 kDa differentiation-associated protein) (Complex I–B17.2) (CI–B17.2) (CIB17.2) (NADH-ubiquinone oxidoreductase subunit B17.2)	48, 49	[214]	Essential component of complex I of the respiratory chain, which transfers electrons from NADH to ubiquinone which is associated with proton pumping out of the matrix [260].
NADH dehydrogenase [ubiquinone] 1 beta subcomplex subunit 10 (Complex I-PDSW) (CI-PDSW) (NADH-ubiquinone oxidoreductase PDSW subunit)	90, 137, 143	[214]	Essential component of complex I of the respiratory chain, which transfers electrons from NADH to ubiquinone which is associated with proton pumping out of the matrix [260].
NADH dehydrogenase [ubiquinone] 1 beta subcomplex subunit 8, mitochondrial (Complex I-ASHI) (CI-ASHI) (NADH-ubiquinone oxidoreductase ASHI subunit)	150, 161, 163, 167	[261]	Essential component of complex I of the respiratory chain, which transfers electrons from NADH to ubiquinone which is associated with proton pumping out of the matrix [260].
NADH dehydrogenase [ubiquinone] 1 beta subcomplex subunit 9 (Complex I–B22) (CI–B22) (LYR motif-containing protein 3) (NADH-ubiquinone oxidoreductase B22 subunit)	39, 97	[214]	Essential component of complex I of the respiratory chain, which transfers electrons from NADH to ubiquinone which is associated with proton pumping out of the matrix [260].
NADH dehydrogenase [ubiquinone] flavoprotein 1, mitochondrial (EC 7.1.1.2) (Complex I-51kD) (CI-51kD) (NADH dehydrogenase flavoprotein 1) (NADH-ubiquinone oxidoreductase 51 kDa subunit)	46, 122	[214]	Essential component of complex I of the respiratory chain, which transfers electrons from NADH to ubiquinone which is associated with proton pumping out of the matrix [260].
NADH dehydrogenase [ubiquinone] iron-sulfur protein 3, mitochondrial (EC 7.1.1.2) (Complex I-30kD) (CI-30kD) (NADH-ubiquinone oxidoreductase 30 kDa subunit)	191, 207, 212	[214]	Essential component of complex I of the respiratory chain, which transfers electrons from NADH to ubiquinone which is associated with proton pumping out of the matrix [260].
NADH dehydrogenase [ubiquinone] iron-sulfur protein 8, mitochondrial (EC 7.1.1.2) (Complex I-23kD) (CI-23kD) (NADH-ubiquinone oxidoreductase 23 kDa subunit) (TYKY subunit)	38, 74, 104, 143	[214]	Essential component of complex I of the respiratory chain, which transfers electrons from NADH to ubiquinone which is associated with proton pumping out of the matrix [260].
NADH-ubiquinone oxidoreductase 75 kDa subunit, mitochondrial (EC 7.1.1.2) (Complex I-75kD) (CI-75kD)	316, 440, 442, 656	[214]	Essential component of complex I of the respiratory chain, which transfers electrons from NADH to ubiquinone which is associated with proton pumping out of the matrix [260].
Peroxiredoxin-1 (EC 1.11.1.24) (Natural killer cell-enhancing factor A) (NKEF-A) (Proliferation-associated gene protein) (PAG) (Thioredoxin peroxidase 2) (Thioredoxin-dependent peroxide reductase 2) (Thioredoxin-dependent peroxiredoxin 1)	116, 194	[262]; [156]; [214]	Peroxide detoxifying enzyme that protects the cell against oxidative damage induced by ROS by thioredoxin peroxidase [263]. Essential in the regulation of cell proliferation and regulating signaling transduction through H_2_O_2_ which acts as a second messenger molecule [264].
Peroxiredoxin-2 (EC 1.11.1.24) (Natural killer cell-enhancing factor B) (NKEF-B) (PRP) (Thiol-specific antioxidant protein) (TSA) (Thioredoxin peroxidase 1) (Thioredoxin-dependent peroxide reductase 1) (Thioredoxin-dependent peroxiredoxin 2)	33, 126, 193	[156]; [262]	Peroxide detoxifying enzyme that protects the cell against oxidative damage induced by ROS by thioredoxin peroxidase [263]. Essential in the regulation of cell proliferation and regulating signaling transduction through H_2_O_2_ which acts as a second messenger molecule [264].
Peroxiredoxin-4 (EC 1.11.1.24) (Antioxidant enzyme AOE372) (AOE37-2) (Peroxiredoxin IV) (Prx-IV) (Thioredoxin peroxidase AO372) (Thioredoxin-dependent peroxide reductase A0372) (Thioredoxin-dependent peroxiredoxin 4)	188, 191, 237	[214]	Peroxide detoxifying enzyme that protects the cell against oxidative damage induced by ROS by thioredoxin peroxidase [263]. Essential in the regulation of cell proliferation and regulating signaling transduction through H_2_O_2_ which acts as a second messenger molecule [264].
Succinate dehydrogenase [ubiquinone] iron-sulfur subunit, mitochondrial (EC 1.3.5.1) (Iron-sulfur subunit of complex II) (Ip)	147, 150, 206, 216, 241	[214]	Dual-role enzyme in TCA and ETC. SDH oxidizes succinate to fumarate and feeds electrons to ubiquinone as Complex II [265].

One illustrative example is the small GTPase RhoA, which undergoes site-specific nitration at Y34 under pathological conditions such as pulmonary hypertension and acute lung injury, where endothelial nitric oxide synthase (eNOS) becomes uncoupled [266]. Nitration at this residue induces constitutive RhoA activation and disrupts endothelial barrier integrity [267]. Extending these findings, it has been shown that nitration-mediated activation of RhoA has been shown to promote mitochondrial fission and enhances glycolytic flux, driving a Warburg-like metabolic phenotype associated with hyperproliferation in pulmonary arterial endothelial cells [101]. Notably, selective shielding of RhoA from nitration using a nitration-inhibitory peptide (Nrp1) reverses these pathological effects [101,267]. Given the central role of aerobic glycolysis in tumor growth and metastasis, these observations identify nitrated RhoA as a compelling yet largely unexplored regulator of cancer metabolism and invasion.

Another candidate class emerging from the nitroproteome is the 14-3-3 family of adaptor proteins, which are highly conserved and ubiquitously expressed regulators of cell cycle progression and signal transduction [268,269]. Through binding to a wide range of client proteins, including transcription factors, kinases, cytoskeletal components, and tumor suppressors, 14-3-3 proteins exert broad control over protein conformation, localization, stability, phosphorylation state, and molecular interactions [268,269]. Tyrosine nitration of 14-3-3β/α at residues Y21, Y106, Y130, and Y213 has been reported [156,214], providing a direct mechanism by which nitrative stress could disrupt these regulatory interactions. In the context of cancer, such modifications could plausibly rewire pro-survival and pro-proliferative signaling networks.

Finally, vimentin, a canonical marker and functional mediator of epithelial-to-mesenchymal transition (EMT), represents another underexplored nitration target. Vimentin is frequently overexpressed in invasive tumors and can be detected at the tumor cell surface in sarcoma, breast cancer, and colon cancer [270]. Multiple tyrosine residues on vimentin, including Y11, Y30, Y117, Y150, Y276, and Y358, have been identified as sites of nitration [156,157,214,271,272]. Given the critical role of vimentin filament dynamics in cytoskeletal remodeling, cell motility, and invasion, nitration-dependent alterations in vimentin structure or assembly may represent a mechanistic link between nitrative stress and EMT-driven tumor progression.

Collectively, these examples underscore the breadth of the cancer nitroproteome and illustrate how site-specific tyrosine nitration of proteins not traditionally viewed as redox regulated can generate functionally distinct proteoforms with the potential to reprogram cellular behavior. Importantly, these candidates are not presented as an exhaustive catalog, but rather as a focused, hypothesis-generating framework.

Together, these observations set the stage for the concluding framework of this review, in which tyrosine nitration is reframed as a selective and regulatory post-translational modification, and the nitroproteome is positioned as a new mechanistic and therapeutic space for target discovery and validation in cancer.

## 11 Conclusion

Reactive nitrogen species (RNS), particularly nitrating species that lead to protein tyrosine nitration, have long been associated with oxidative injury. The evidence reviewed here supports a more transformative interpretation of their role in cancer: in addition to the well-established oxidative modifications that target cysteine residues, protein tyrosine nitration emerges as a selective, site-specific post-translational modification that functions as a regulatory determinant of cancer signaling, not merely a byproduct of nitrative stress.

A defining feature of tyrosine nitration is its restricted target landscape. Rather than occurring randomly, nitration modifies discrete tyrosine residues within proteins positioned at critical signaling and metabolic nodes. These modifications generate functionally distinct nitrated proteoforms with suppressed, enhanced, or reprogrammed activity depending on structural context. In this regard, nitration operates as a redox-dependent signaling modality that parallels phosphorylation in its capacity to modulate protein function yet differs fundamentally in that it is driven by inflammatory and metabolic redox flux rather than enzymatic kinase cascades. Through selective residue modification, nitration rewires phosphorylation-dependent pathways, alters protein-protein interactions, reshapes chaperone and phosphatase activity, redirects metabolic flux, and modulates immune signaling.

This selectivity confers regulatory power. By targeting proteins that govern proliferation, apoptosis, angiogenesis, invasion, metabolic plasticity, and immune surveillance, tyrosine nitration provides tumor cells with a mechanism to translate chronic inflammation and redox imbalance into durable changes in cellular phenotype. These findings challenge the historical view of nitration as a passive marker of oxidative stress and instead position it as an active, context-dependent driver of oncogenic signaling plasticity.

Reframing the ensemble of nitrated proteins as the cancer nitroproteome defines a new mechanistic and therapeutic landscape. Nitrated proteins arise under pathological conditions and frequently exhibit gain- or loss-of-function activities that are not present in their unmodified counterparts, thereby creating disease-restricted molecular states that may be selectively targeted. The identification of site-specific nitration events provides a framework for target validation, biomarker discovery, and therapeutic development. These include strategies to prevent critical nitration events, shield vulnerable residues, selectively degrade nitrated proteoforms, exploit nitration-induced functional dependencies, or identify nitrated proteins displayed on the tumor cell surface that could serve as neoantigens for CAR-T cell-based immunotherapies. In parallel, modulation of intratumoral RNS to reverse nitration-dependent immune suppression offers an additional axis for combination with immunotherapy.

As nitroproteomic technologies, structural analyses, chemical biology platforms, and disease models continue to advance, systematic interrogation of nitration events will be essential to distinguish causal regulatory modifications from secondary oxidative changes. The model that emerges is one in which inflammatory RNS generate selective tyrosine nitration at defined signaling nodes, producing nitrated proteoforms that reprogram cancer cell behavior and create therapeutically exploitable vulnerabilities.

Collectively, the evidence positions protein tyrosine nitration as a selective and regulatory post-translational modification that encodes redox information into cancer signaling networks and establishes the nitroproteome as a novel and actionable frontier for mechanistic discovery and precision oncology.

## CRediT authorship contribution statement

**Inge Claassen:** Data curation, Investigation, Visualization, Writing – original draft, Writing – review & editing. **Nirbachita Adrita:** Writing – review & editing. **Kevin B. Chandler:** Writing – review & editing. **Stephen M. Black:** Writing – review & editing. **Blaine R. Roberts:** Writing – review & editing. **Alvaro G. Estevez:** Writing – review & editing. **Joseph S. Beckman:** Writing – review & editing. **Maria Clara Franco:** Conceptualization, Funding acquisition, Project administration, Resources, Supervision, Visualization, Writing – review & editing.

## Declaration of competing interest

The authors have no competing interests.

## Data Availability

No data was used for the research described in the article.
